# Can Pre-Contraction of the Pelvic Floor Muscles Exceed Increases in Intra-Abdominal Pressure During Strength Exercises?

**DOI:** 10.1007/s00192-026-06540-8

**Published:** 2026-02-28

**Authors:** Clara Bjurulf, Lingge Meng, David Budgett, Jennifer Kruger, Kari Bø

**Affiliations:** 1 The Norwegian School of Sport Sciences, Department of Sports Medicine, Ullevål Stadion, Sognsveien 220, 0806 Oslo, Norway; 2 Auckland Bioengineering Institute, University of Auckland, Auckland, New Zealand

**Keywords:** Feasibility, Pelvic floor muscles, Physiotherapy, Precontraction, Prevention, Rehabilitation

## Abstract

**Introduction and Hypothesis:**

A recent study found that young power and weightlifters reported a high prevalence of pelvic floor disorders (PFDs) with urinary incontinence (UI) 50%, anal incontinence 80% and pelvic organ prolapse 23%. Performing “the knack”, a voluntary pre-contraction of the pelvic floor muscles (PFMs), before and during stressful events, can prevent UI during coughing and daily activities. However, the effect of this manoeuvre has not been investigated during strenuous exercises. This study was aimed at investigating the feasibility of using the femfit® to measure intra-abdominal pressure (IAP) and PFM pressure during strength exercises and at discovering if a voluntary pre-contraction of the PFMs can exceed increases in IAP during strength exercises.

**Methods:**

This was a short-term, cross-sectional, experimental study. Eleven participants were tested in squat, deadlift, leg press and curl up, performed in a random order with and without voluntary pre-contraction of the PFMs. Assessment of the ability to contract the PFMs was conducted by suprapubic 2D ultrasound. IAP and PFM pressure were measured with the femfit®. Wilcoxon Signed Rank Test was used to estimate differences in change between IAP and PFM pressure with and without voluntary pre-contraction during the strength exercises.

**Results:**

No participants reported displacement or discomfort from the femfit® device. Voluntary pre-contraction of the PFMs did not exceed the rise in IAP during squat, deadlift, leg press and curl up.

**Conclusions:**

The PFM pressure was not significantly higher than the IAP during strength exercises. Further longitudinal studies are warranted to investigate if systematic PFM training can improve the strength of the voluntary pre-contraction.

## Introduction

Strength training, including heavy weightlifting, has become increasingly popular among women [1, 2]. A recent study found that young power- and Olympic weightlifters reported a high prevalence of pelvic floor disorders (PFDs) with urinary incontinence (UI) affecting 50%, anal incontinence (AI) 80% and pelvic organ prolapse (POP) 23% [3].

Known risk factors for PFDs in the general population include older age, obesity, pregnancy and vaginal childbirth, with injuries to the connective tissue, peripheral nerves, pelvic floor muscles (PFMs) and perineum [4]. The underlying mechanisms of PFDs in strength athletes have not yet been established. Strenuous exercise and heavy lifting lead to increases in intra-abdominal pressure (IAP) that may result in downward pressure on the pelvic floor. To date, there are two conflicting hypotheses regarding the effect of IAP during exercise on the pelvic floor [5]. The PFMs may be strengthened owing to possible training adaptations through the stretch and fatigue of the PFMs and/or a co-contraction of the PFMs. In contrast, if the PFMs are not able to counteract increases in IAP, the pelvic floor may be stretched, overloaded and weakened and may thereby increase the risk of PFDs [5].

A pre-contraction described as a voluntary contraction of the PFMs timed before and during stressful events, known as “the knack”, has been shown to reduce urinary leakage in the general female population by 73–98% [6]. Basic research studies have demonstrated that performing a voluntary pre-contraction stabilises the pelvic floor and significantly reduces bladder-neck descent [7, 8]. However, its efficiency has not yet been evaluated during heavy lifts and strenuous strength training.

Various tools are available to assess different aspects of PFM function. However, measuring PFM function during physical activity has been challenging because existing tools have not been able to distinguish between PFM activity and increases in IAP. The femfit®, a relatively new intravaginal pressure device, has been shown to reliably measure PFM contraction and IAP during physical activity [9]. Moderate to high correlation of assessment of PFM strength has been found with a dynamometer, by palpation and with ultrasound. However, the femfit® has not yet been tested during strenuous strength exercises. The aim of the present study is to evaluate the feasibility of using femfit® to measure IAP and PFM pressure during strength exercises, and to investigate if a voluntary pre-contraction of the PFMs can exceed increases in IAP during strength training.

## Materials and Methods

### Design

A short-term, cross-sectional, experimental design was used to answer the research questions. The data were collected at one occasion for each participant at a sports university laboratory from 21 October to 23 November 2024. Participants were tested in four exercises (squat, deadlift, leg press and curl up) and each exercise was performed with and without voluntary pre-contraction of the PFMs. Following a random allocation sequence made by a statistician, the participants were randomised to start the exercises either with or without voluntary pre-contraction, before performing the exercises again, but in reverse order. To secure standardisation of performance, instructional videos with verbal cues guided the participants when and how to perform the different exercises. The data collection was conducted by one person only; a physiotherapist trained to use the measurement methods of the study. The physiotherapist instructed, followed and monitored all exercises to ensure proper technique.

### Participants

Eleven participants were recruited through announcements at the University of Sport Science, through word of mouth, and on social media through the Physiotherapy Association Subgroup of Women’s Health. Inclusion criteria were physically active women aged between 18 and 35 years, performing strength training ≥ once/week and able to lift their own body weight for two repetitions in squat, deadlift and leg press. Exclusion criteria were males, age below 18 and above 35 years, pregnancy, inability to contract the PFMs and injuries or surgeries that could affect participation or results.

The study was approved by the Regional Committees for Medical and Health Research Ethics (Reference number: 762897/REK sør-øst D), and storage of data was approved by the Agency for Norwegian Agency for Shared Services in Education and Research (SIKT) (Reference number: 339980). All participants signed informed consent before the start of the study. The University of Sport Sciences was responsible for ensuring that the transfer of data complied with the country’s law and the EU’s General Data Protection Regulation.

### Background Data

The researcher measured the height (Seca 213, PorTable height measure) and weight (Seca 869, Flat scale with cable remote display) of each participant and calculated BMI by using the formula weight(kg)/height(m)^2^ [10]. An online questionnaire (for questions see Table 1) was used to collect background variables (age, education, exercise exposure) and health status (births, chronic disease, injury, pain, surgeries and specific questions on PFDs). Standardised questionnaires from the International Consultation on Incontinence Questionnaire (ICIQ) were used to assess PFDs [11]. Ability to contract the PFMs was assessed by two-dimensional transabdominal ultrasound (GE Healthcare Logiq e R7, GE > 12L-RS—5–13 MHz Wideband Linear Probe), with the probe placed suprapubically.

**Table 1:** Background variables, training experience and health status

	All participants (*N* = 11)
Age (years), mean (SD, minimum–maximum)	25.6 (4.2, 18–32)
BMI, mean (SD, minimum–maximum)	24.6 (1.6, 23–28)
Students, *n* (%)	
High school	2 (18.2%)
Bachelor	4 (36.4%)
Master	5 (45.5%)
Years of strength training, mean (SD, minimum–maximum)	8.2 (2.4, 3–10)
Strength training days/week, *n* (%)	
1–3 days	7 (63.6)
4–5 days	4 (36.4)
Duration of strength training minutes per session, mean (SD, minimum–maximum)	73.6 (15.7, 60–90)
Type of strength training, *n* (%)	
Weightlifting	4 (36.4)
Powerlifting	1 (9.1)
Functional strength	4 (36.4)
CrossFit	1 (9.1)
Others	1 (9.1)
Time per week on other sports (hours of training/week) mean (SD, minimum–maximum)	5.1 (3.1, 2–12)
Parity, *n* (%)	
0	10 (90.9)
1	1 (9.1)
Chronic diseases, *n* (%)	
Asthma	1 (9.1)
Injuries/pain *n* (%)	
Back	3 (27.3)
Stomach	1 (9.1)
Hip	4 (36.4)
Pelvis	0 (0)
Coccyx	0 (0)
Surgeries, *n* (%)	
Abdominal hernia	1 (9.1)
Prevalence UI daily life, *n* (%)	
Never	6 (54.5)
One time/weak or less	4 (36.4)
2–3 times/week	1 (9.1)
One time/day	0 (0)
Several times/day	0 (0)
All the time	0 (0)
Amount of urine loss, *n* (%)	
Nothing	5 (45.5)
A small amount	6 (54.5)
A moderate amount	0 (0)
A large amount	0 (0)
Influence on daily life (scale from 0–10)	
0	7 (63.6)
1	2 (18.2)
2	1 (9.1)
3	1 (9.1)
Prevalence of UI during training/competition, *n* (%)	
Never	5 (45.5)
Rarely	5 (45.5)
Sometimes	1 (9.1)
ICIQ-UI SF sum score, mean (SD)	2.1 (2.4)
Prevalence of POP symptoms in daily life, *n* (%)	0
Prevalence of POP symptoms during training/competition, *n* (%)	0
Prevalence of AI in daily life, *n* (%)	
Loose stool	1 (9.1)
Solid stool	0
Wind	5 (45.5)
Prevalence of AI during training/competition, *n* (%)	
Loose stool	0
Solid stool	0
Wind	5 (45.5)

*SD* standard deviation, *BMI* body mass index, *UI* urinary leakage, *ICIQ-UI SF* International Consultation on Incontinence Questionnaire—Urinary Incontinence Short Form, *POP* pelvic organ prolapse, *AI* anal incontinence

### Participants’ Experience with femfit®

After each exercise the participants were asked an a priori set of questions related to the feasibility of the device; if they perceived any discomfort or displacement of the pressure sensor array, whether they could maintain the voluntary pre-contraction throughout the exercise, if they perceived PFM fatigue and if they experienced symptoms of PFDs during the exercises. Possible responses were: “Yes”, “No” or “I don’t know”. Also, acceptable signal loss and displacement were considered a priori and registered.

### Testing transcript and instructional videos

The four exercises were divided into two sessions. In the first session, squat and deadlift were recorded and in the second session leg press and curl up were recorded. Four testing transcripts were created, two per session (starting either with or without voluntary pre-contraction). The transcripts were identical for each session (except for the order of voluntary pre-contraction) to promote standardised timing and execution of the different activities. Based on the testing transcript, four instructional videos were developed and instructed the participants when and how to perform the exercises. The instructions were provided verbally, in written form and through a demonstration of the exercises in the video. Resting periods and maximal voluntary contractions (MVCs) of the PFMs were implemented in the testing transcript to facilitate data extraction for the data analysis. The sessions were recorded by program 70 in the femfit® app, which saved data continuously for 7 minutes.

### The femfit® Measurements

**Fig. 1:**
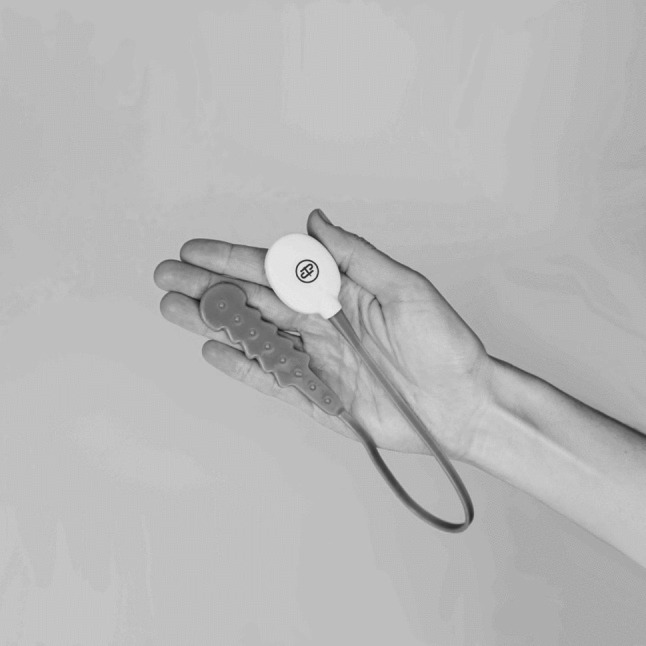
The femfit® device, showing the pressure sensor array and the pod used for Bluetooth connection. Image used with permission from Junofem Ltd

The IAP and PFM pressure were measured using femfit®, an intravaginal sensor array measuring a pressure profile at eight locations along the length of the vagina [9]. For each participant, the IAP value was calculated using data from the deepest sensor (sensor 8, located beyond the PFM region), by determining the difference between the resting IAP prior to the exercise and the highest pressure recorded during the activity. The sensor (between 1 and 7, located at the level of the PFMs) with the highest (peak) pressure during the three MVCs of the PFMs performed before each exercise was used to represent the PFM pressure during the subsequent exercise. The PFM pressure value was then calculated using the same method as used for IAP. We use the term PFM pressure with and without voluntary pre-contraction during the four strength exercises as opposed to the three MVCs of the PFMs performed before the experiment. IAP and PFM pressure were measured in millimetres of mercury (mmHg; Fig. 1).

### Procedure

After receiving written information about the study, reviewing inclusion and exclusion criteria, and agreeing to participate, women completed the informed consent form. Participants’ height and weight were measured, and they answered the questionnaire about background variables, health status and specific questions on PFDs. The researcher used an anatomy app (Essential Anatomy 5 app, 3D4Medical) to teach each participant about the pelvic floor and how to perform a correct voluntary contraction before assessing the ability to contract the PFMs. A correct voluntary PFM contraction was described as an elevation and squeeze around the urethra, vagina and rectum [12]. For the first attempt, the participants were instructed to sit on the edge of the bench or armrest to feel the pressure against the perineum. The participants were given the following instructions: “Pull up and in around the urethra, vagina and anus, as if you are trying to lift your pelvic floor off the bench/armrest”. The researcher asked how it felt and whether the participants felt like they were able to perform a voluntary contraction of the PFMs. Thereafter, ability to contract the PFMs was assessed as a bladder base elevation using 2D transabdominal ultrasound with the participants in a supine crook-lying position. Only participants able to contract were included in the study. Instructions on how to clean and insert the femfit® device were shown through the femfit® app, and the participants cleaned and inserted the device into the vagina in a private room. Information about randomised order of contraction were given and the participants were shown instructional videos in the selected randomised order. The participants practiced the protocol and warmed up by following the instructional video, while lifting gradually heavier weights.

### Statistical Methods

Background variables, including prevalence of UI, AI and POP, were described with mean and standard deviation or numbers with percentages. The results of the experiment are presented as median with interquartile range (IQR) and 95% confidence intervals. Wilcoxon signed rank test was used to estimate differences in change in pressure profiles between the IAP and PFMs with and without voluntary pre-contraction of the PFMs. As this is a pilot, exploratory study, an a priori power calculation was not conducted.

## Results

Twenty-six women were assessed for eligibility. Five women did not meet the inclusion criteria, threewere unable to lift sufficiently heavy weights in the squat and two were not able to contract the PFMs. Four women declined to participate owing to time constraints. Injuries (meniscus rupture, disc herniation, stress fracture and a painful foot) prevented four women from participating in the study. Two women became pregnant and were thereby excluded from participation. Eleven women were included in the study; five participants performed the exercises without voluntary pre-contraction first and then with voluntary pre-contraction, and six participants performed the exercises with voluntary pre-contraction first and then without. None of the eleven participants was excluded from the data analysis. The randomised order of voluntary pre-contraction did not have a significant effect on the overall results.

Table 1 presents background variables, training experience and health status (including PFDs). The participants had a mean age of 25.6 years (SD ± 4.3), a mean BMI of 24.6 (SD ± 1.6) and a strength training frequency of 1–3 times per week with a mean of 70 minutes per session. Most of the participants trained with either weightlifting or by doing functional strength training. They also engaged in additional sports, including running, cycling, handball, volleyball and combat sports. None had previously undertaken systematic strength training of the PFMs.

The participants were healthy, with no history of injuries or pain in the pelvic or coccygeal region. Most participants were nulliparous. Almost half of the participants reported experience of UI and AI (losses of wind) both during daily life and during sport performance. None reported symptoms of POP. The participants who experienced UI reported a small amount of leakage, which occurred once a week or less frequently. The impact on daily life was minimal and mean sum score on the ICIQ-Urinary Incontinence Short Form (ICIQ-UI SF) indicated no or minimal UI symptoms.

Table 2 presents the participants´ answers regarding the feasibility of using the femfit® device to measure IAP and PFM pressure during strength exercises. No displacement or discomfort during testing was reported. One participant reported perceiving some movement of the device, although this was not considered sufficient to warrant repositioning. None of the participants experienced symptoms of PFDs during the experiment. The squat was identified as the most challenging exercise for maintaining a voluntary pre-contraction of the PFMs, followed by the deadlift. Most participants reported being successful in voluntary pre-contracting the PFMs during the leg press and curl-up. Few participants reported PFM fatigue during the testing. Occasionally, there was signal loss during the leg press and curl up, likely due to interference of the Bluetooth signal when the pod slipped between the hip and thigh. Participants were then asked to reposition the pod, and the session was re-recorded. No other issues were encountered.

**Table 2:** Participants answer with regard to the feasibility of the femfit® device for measuring intra-abdominal pressure (IAP) and pelvic floor muscle (PFM) pressure during strength exercises

Question	Data
Did you feel any movement or displacement of the femfit® device?	
Yes	1 (9.1)
No	10 (90.9)
Maybe	0 (0)
Did the femfit® device provoke discomfort?	
Yes	0 (0) 0 (0)
No	11 (100)
Maybe	11 (100)
Did the exercise provoke leakage?	
Yes	0 (0)
No	11 (100)
Maybe	0 (0)
Did the exercise provoke downward pressure/vaginal bulge?	
Yes	0 (0)
No	11 (100)
Maybe	0 (0)
Did you manage to hold the PFM contraction during the squat?	
Yes	2 (18.2)
No	4 (36.4)
Maybe	5 (45.5)
Did you manage to hold the PFM contraction during the deadlift?	
Yes	4 (36.4)
No	3 (27.3)
Maybe	4 (36.4)
Did you manage to hold the PFM contraction during the leg press?	
Yes	10 (90.9)
No	1 (9.1)
Maybe	0 (0)
Did you manage to hold the PFM contraction during the curl up?	
Yes	9 (81.8)
No	0 (0)
Maybe	2 (18.2)
Did you perceive PFM fatigue during the testing?	
Yes	2 (18.2)
No	7 (63.6)
Maybe	2 (18.2)

Values are presented as numbers of participants and percentage of the sample

*IAP* intra-abdominal pressure, *PFM* pelvic floor muscle

Tables 3 and 4 present the peak pressure values of three MVCs of the PFMs for each of the participants before the four strength exercises and the pressure rise of the PFMs and IAP with and without a voluntary pre-contraction during the strength exercises. During the three MVCs of the PFMs, the pressure ranged between 0.8 and 31.1 mmHg, whereas IAP ranged between −0.2 to 20.7 mmHg. Before the four strength exercises, most participants showed greater pressure during PFM contraction than increases in IAP. There were variations between participants across the four strength exercises, but participants generally generated both higher automatic co-contraction and voluntary contraction of the PFMs and IAP during squat and deadlift than during leg press and curl up.

**Table 3:** Peak pressure (mmHg) of three maximum voluntary contractions (MVCs) of the pelvic floor muscles (PFMs) before squat and deadlift, and PFMs and intra-abdominal pressure (IAP) during squat and deadlift, with and without voluntary pre-contraction (PreC), for each participant

Participants	Peak PFM pressure of three MVCs before squat	Peak IAP of three MVCs before squat	PFM pressure during squat without PreC	PFM pressure during squat with PreC	IAP during squat without PreC	IAP during squat with PreC	Peak PFM pressure of three MVCs before deadlift	Peak IAP of three MVCs before deadlift	PFM pressure during deadlift without PreC	PFM pressure during deadlift with PreC	IAP during deadlift without PreC	IAP during deadlift with PreC
1	14.3	3.9	24.8	17.9	52.2	48.0	11.4	7.4	19.8	21.8	24.3	23.8
2	5.1	2.4	13.7	5.1	17.4	28.3	0.8	0.7	44.8	49.0	45.0	54.4
3	24.5	7.6	39.8	34.8	53.4	41.4	18.6	10.2	36.6	45.1	17.4	19.3
4	14.8	13.2	51.5	56.1	47.8	50.0	21.7	20.7	36.2	54.4	30.9	52.0
5	7.7	5.7	24.7	33.2	46.4	49.7	11.7	2.8	8.3	13.0	11.4	12.1
6	11.2	7.3	20.0	17.7	15.3	14.3	12.1	7.9	14.8	24.7	11.5	19.1
7	9.1	8.8	34.3	45.0	39.9	58.3	8.9	8.9	−0.3	2.4	20.2	42.8
8	19.7	15.7	59.3	59.6	45.4	50.4	19.7	12.2	31.7	48.6	37.0	25.3
9	10.1	4.8	31.2	43.3	37.6	36.4	10.3	4.7	34.1	41.1	33.5	46.6
10	11.4	3.2	11.8	17.7	5.2	17.5	11.2	4.2	6.4	8.1	3.8	16.2
11	11.5	8.0	74.5	63.0	65.0	65.5	11.7	7.2	47.2	48.8	34.9	36.7

**Table 4:** Peak pressure (mmHg) of three maximum voluntary contractions (MVCs) of the pelvic floor muscles (PFMs) before leg press and curl up, and PFMs and intra-abdominal pressure (IAP) during leg press and curl up, with and without voluntary pre-contraction (PreC), for each participant

Participants	Peak PFM pressure of three MVCs	Peak IAP of three MVCs	PFM pressure during leg press without PreC	PFM pressure during leg press with PreC	IAP during leg press without PreC	IAP during leg press with PreC	Peak PFM pressure of three MVCs	Peak IAP of three MVCs	PFM pressure during curl up without PreC	PFM pressure during curl up with PreC	IAP during curl up without PreC	IAP during curl up with PreC
1	7.6	1.7	11.5	12.8	4.4	13.3	11.4	.9	6.9	11.4	4.3	6.6
2	7.5	1.5	0.4	3.4	6.8	10.6	6.4	−0.2	11.7	10.0	11.7	13.9
3	31.1	0.5	36.7	54.3	3.8	5.0	27.9	2.2	0.9	30.2	3.7	3.9
4	2.3	1.4	5.4	36.3	6.0	34.0	7.7	1.9	7.4	35.4	5.5	29.7
5	9.7	2.8	10.5	20.5	2.9	20.8	14.9	3.3	5.9	14.8	3.8	6.7
6	2.0	0.9	5.5	10.2	3.0	4.8	1.6	0.1	5.4	5.9	2.0	2.7
7	0.9	2.6	2.5	3.1	10.3	5.7	6.3	3.8	4.2	6.7	6.2	2.7
8	10.8	4.8	6.9	34.4	2.7	21.4	5.9	2.4	8.2	16.4	4.0	11.4
9	17.1	1.6	31.9	55.2	6.2	17.7	18.5	0.9	4.9	14.3	6.2	9.6
10	0.9	0.6	2.0	7.1	1.0	5.9	0.9	0.6	9.3	7.2	7.5	5.5
11	14.8	2.1	9.2	14.8	2.8	1.9	8.0	3.1	8.5	5.4	15.1	11.8

### Can a voluntary pre-contraction of the PFMs (performing the knack) exceed increases in IAP during four strength exercises?

**Fig. 2:**
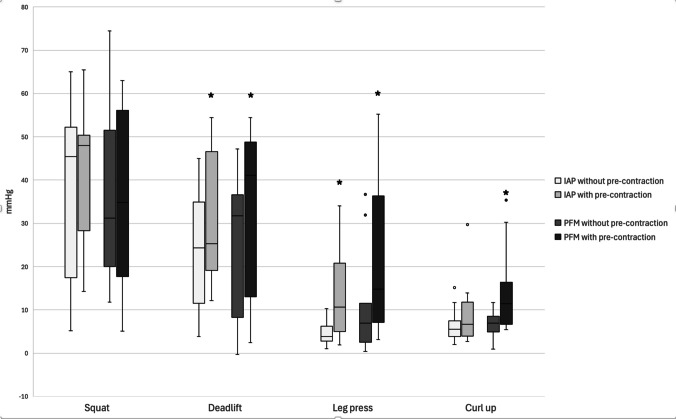
Increases in intra-abdominal pressure (IAP) and pelvic floor muscle (PFM) pressure in strength exercises with and without voluntary pre-contraction of the PFMs (median with interquartile range). IAP = intra-abdominal pressure, PFM = pelvic floor muscle, * = *p* < 0.05

Figure 2 presents the increases in IAP and PFM pressure in the four strength exercises with and without voluntary pre-contraction of the PFMs. Both IAP and PFM pressure increased significantly with voluntary pre-contraction in most exercises, except for the squat, and IAP during the curl up. The increase in PFM pressure did not significantly exceed the increases in IAP during any of the exercises with voluntary pre-contraction. Table 5 shows details of point estimates and variation.

**Table 5:** Difference between pelvic floor muscle (PFM) pressure and intra-abdominal pressure (IAP) during the four strength exercises, with and without voluntary pre-contraction of the PFM (performing “the knack”)

Exercises	PFM pressure			IAP				
	Median (IQR)	95% CI	Mean ± SD	Median (IQR)	95% CI	Mean ± SD	Median differences	*p* value
Squat without voluntary pre-contraction	31.2 (20.0–51.5)	13.7, 59.3	35.1 ± 19.7	45.4 (17.4–52.2)	15.3, 53.4	38.7 ± 18.4	−14.2	0.534
Squat with voluntary pre-contraction	34.8 (17.7–56.1)	17.7, 59.6	35.8 ± 19.5	48.0 (28.3–50.4)	17.5, 58.3	41.8 ± 16.2	−13.2	0.286
Deadlift without voluntary pre-contraction	31.7 (8.3–36.6)	8.3, 44.8	25.4 ± 16.3	24.3 (11.5–34.9)	11.4, 37.0	24.5 ± 12.8	7.4	0.790
Deadlift with voluntary pre-contraction	41.1 (13–48.8)	8.1, 49.0	32.5 ± 18.9	25.3 (19.1–46.6)	16.2, 52.0	31.7 ± 15.3	15.8	0,722
Leg press without voluntary pre-contraction	6.9 (2.5–11.5)	2.0, 31.9	11.1 ± 12.2	3.8 (2.8–6.2)	2.7, 6.8	4.5 ± 2.6	3.1	0.110
Leg press with voluntary pre-contraction	14.8 (7.1–36.3)	3.4, 54.3	22.2 ± 19.2	10.6 (5.0–20.8)	4.8, 21.4	12.8 ± 9.8	4.2	0.110
Curl up without voluntary pre-contraction	6.9 (4.9–8.5)	4.2, 9.3	6.7 ± 2.9	5.5 (3.8–7.5)	3.7, 11.7	6.4 ± 3.9	1.4	0.594
Curl up with voluntary pre-contraction	11.4 (6.7–16.4)	5.9, 30.2	14.3 ± 9.9	6.7 (3.9–11.8)	2.7, 13.9	9.5 ± 7.7	4.7	0.062

Values are shown in mmHg

*IQR* interquartile range, *CI* confidence interval, *SD* standard deviation

## Discussion

Our findings suggest that it was feasible to use the femfit® device to measure IAP and PFM pressure during strength exercises without provoking discomfort or displacement of the femfit® device in any of the young, healthy and active participating women. Voluntary pre-contraction of the PFMs (performing the knack) led to significantly higher IAP and PFM pressure during deadlift, leg press and curl up than exercises performed without voluntary pre-contraction, but not during the squat or in IAP during the curl up. However, our results showed that voluntary pre-contraction of the PFMs did not significantly exceed increases in IAP during any of the four strength exercises. Although commonly used and advocated, voluntary pre-contraction of the PFMs has to our knowledge not previously been investigated during strenuous strength exercises.

### Feasibility of the femfit®

The femfit® device is a relatively new measurement tool for assessing PFM function [9]. Currently available tools assess different aspects of PFM function and none of them can simultaneously assess pressure from the PFMs and IAP [13, 14]. Cacciari et al. [9] tested the femfit® during PFM contractions and the Valsalva manoeuvre in standing and lying positions in 20 healthy women. They found an average intraclass correlation of intra- and intersession reliability of 0.98 in lying and 0.78 in standing within sessions.

In the present study, one of the eleven participants reported feeling movement of the device during the leg press, but none reported displacement. Cacciari et al. [9] reported that the femfit® device required repositioning after straining manoeuvres on six occasions in lying and standing positions, although the device did not fall out. The mean IAP was 18.9 ± 11.3 mmHg (range = 3.9–56 mmHg) during straining, with the higher range being above the IAP measured during squat in the present study (52.2 mmHg). We did not check the position of the femfit® device after testing and had to rely on the participants reports and the measurements shown through the femfit® app. Cacciari et al. [9] did not report any signal loss or measurement error in their reliability and validity study. However, they assessed PFM contraction and straining only in standing and lying positions, with no measurements performed during dynamic movement.

### Pelvic Floor Muscle Maximum Voluntary Contraction and Intra-Abdominal Pressure Before Commencing the Four Strength Exercises

Pelvic floor muscle strength was relatively weak (ranging from 7.6 to 11.7 mmHg, depending on the position of the strength exercise), whereas increases in IAP were relatively high (ranging from 1.6 to 7.4 mmHg, also depending on the position). Additionally, there was considerable variation in participants’ ability to perform MVCs without simultaneously increasing IAP. Kruger et al. [15] used the femfit® device to measure PFM contractions in 19 healthy adults and reported a mean of 16.3 ± 12.2 mmHg with simultaneous IAP values of 3.4 ± 2.2 mmHg. Interestingly, some participants demonstrated PFM strength comparable with women with PFDs [16, 17]. Similarly, Hagovská et al. [18] found no significant difference between healthy women and women with SUI using the femfit®. The participants in the present study were healthy and had a mean score of 2.1 ± 2.4 on the ICIQ-UI SF and no-one leaked during the experiment. Although the average score indicated no or minimal UI symptoms, nearly half reported experience of UI and AI (wind) in daily life and/or during training or competition. Participants were instructed to contract the PFMs as hard as possible during the three MVCs in the instructional videos. It is possible that IAP would have been lower if additional instructions had emphasised avoiding contraction of nearby muscles and if the participants had trained the PFMs over time.

### Voluntary Pre-Contraction of the Pelvic Floor Muscles (Performing the Knack)

The main aim of the present study was to assess whether a voluntary pre-contraction, performing the knack, exceeded the rise in IAP during the four strength exercises. The results showed that the voluntary pre-contraction did not significantly exceed increases in IAP during any of the exercises. The knack was originally developed for a single task such as a cough to prevent urinary leakage [19]. EMG and perineal ultrasound have shown significantly less bladder neck descent during a cough (4.7 ± 2.9 mm) with voluntary pre-contraction in nulliparous women, than during a cough without voluntary pre-contraction (8.1 ± 2.9 mm) [8]. However, as far as we have ascertained, the effect of the knack on IAP has not previously been assessed. In the present study, we only investigated the immediate effect of using the knack during four exercises, as recommended for elite and strenuous exercisers, and did not study the effect of training voluntary pre-contraction over time. Miller et al. [19] found that women with SUI could acquire the skill of using a timed voluntary pre-contraction to significantly reduce urine leakage during a cough within one week of practice. In a more recent study, 71% of women with SUI and UUI reported significant improvement one month after watching a knack tutorial, compared with 25% in the control group [6].

The participants in the present study had never systematically strength trained the PFMs and were instructed on how to perform a PFM contraction shortly before the testing procedure. We hypothesise that strength training of the PFMs may be necessary to perform an effective voluntary pre-contraction during strenuous strength exercises. This, however, must be evaluated in a randomised controlled trial.

The squat was identified as the most challenging exercise for maintaining a voluntary pre-contraction of the PFMs. As participants in the present study lifted their own body weight, the squat was the heaviest exercise among the exercises included. This finding is consistent with reported risk factors, as exercises involving heavier loads and specific exercises are known to provoke UI in strength athletes [19, 20]. A stronger voluntary pre-contraction held for a longer duration might be necessary to exceed IAP during strength exercises performed under maximum load compared with a cough. Although a cough is a single task that lasts about a second, strength exercises involve managing heavy external loads and maintaining a proper technique, which requires dual tasking [21]. Hence, using the knack during short, reflexive tasks (e.g. coughing) may be fundamentally different when concerning the neuromuscular and cognitive demands of sustained, high-load strength exercises. Further studies are warranted to investigate whether it is possible to achieve a sufficiently strong voluntary pre-contraction during strenuous strength training near maximum load after systematic strength training of the PFMs. An interesting question is whether only some counteraction of the IAP would be beneficial, and additionally, how the counteraction translates to pelvic-floor decent.

The participants in the present study were healthy, and no symptoms of PFDs were reported during the testing. Our results may not extrapolate to women with clinically significant or exercise-induced PFDs. Further research including women with symptoms of PFDs during strenuous strength exercises are warranted to achieve a better understanding of the relationship between IAP and PFMs, and to investigate if a voluntary pre-contraction can be used as prevention in this population.

### Strength and Weaknesses of the Study

One strength of the study is the assessment of the feasibility of the femfit® device for simultaneously measuring IAP and PFM pressure during strength exercises. Many women have trouble contracting their PFMs without straining or engaging other muscle groups [12, 22]. Therefore, each participant had to demonstrate a correct PFM contraction before enrolment. Participants were taught how to perform a PFM contraction using transabdominal ultrasound, during which clear instructions were provided, and real-time biofeedback was used to correct the technique if needed. Each measurement during the four exercises was preceded by assessment of three MVCs of the PFMs for comparison. Instructional videos guided the participants on when and how to perform the exercises, and were essential for ensuring accurate timing for data extraction. In addition, a standardised approach to selecting sensors was used to maintain consistency across exercises.

No a priori power calculation was conducted, as this was an exploratory pilot study and there were no comparable studies on which to base the calculation. However, the results of the present study can be used for future a priori power calculations. The small sample size and the homogenous group of women limit generalisability, which represents a major limitation. Further and larger studies are needed to confirm these preliminary results. It would be interesting to use the device on women with different characteristics, e.g., exercise experience, age, postpartum, etc. The Wilcoxon Test was used for statistical analysis. A non-parametric test has lower statistical power than a parametric test, and is therefore less likely to detect a statistically significant change [23, 24]. To standardise the weight lifted in each exercise, the inclusion criterion for this study was the ability to lift one’s body weight for squat, deadlift and leg press. This method has not been validated as a reliable measure of strength. The one-repetition maximum (1RM) is defined as “the maximal weight that can be lifted once with correct lifting technique” [25, 26], and has demonstrated good to excellent test–retest reliability, regardless of training experience, exercise selection, body part, sex and age of participants [27]. However, 1RM was not tested owing to the design and scope of the present study. The femfit® measures a pressure profile along the length of the vagina, and in the present study the result is either due to an automatic co-contraction (with no voluntary contraction of the PFMs) or due to a voluntary contraction. In practice, the recorded pressures may be influenced by anatomical variations and IAP. There are, however, eight pressure sensors along the length of the pressure sensor array to account for anatomical variability. Changes in the vaginal pressure profile during dynamic exercise is methodically challenging, hence the meticulous attention to detail in defining the methodology in the present study. PFM strength was recorded during three MVCs prior to the four exercise tasks to locate the sensor that measured the highest (peak) pressure. This was designated as the PFM sensor. Sensor 8 located at the top of the vagina (above the levator plate) is well accepted anatomically as a surrogate measure for IAP. Therefore, during the four exercise tasks, the measured PFM pressure was very likely due to the activation of the PFMs acting on the vaginal walls in that region, whereas sensor 8 reflected IAP. Each sensor measures pressure independently of the others.

## Conclusion

This study demonstrated that the femfit® device is a feasible tool for measuring IAP and PFM pressure during strength exercises without provoking discomfort or displacement of the femfit® device. The PFM pressure was not statistically significantly higher than the IAP during squat, deadlift, leg press or curl up. Therefore, based on our results, we cannot recommend strength athletes to contract the PFMs to exceed the IAP. Caution should be taken owing to the small number of participants in the study. Further studies are warranted to investigate if systematic strength training of the PFMs can improve the strength of voluntary pre-contraction during strength exercises.

## Data Availability

The data is to be stored at the Norwegian School of Sport Sciences for 5 years after the end of the study, and may be available on request.

## Appendix 1: Testing Transcript—Overview of the Overall Structure

**Table:**

Session 1: squat and deadlift • 5-second resting period • 1 PFM contraction • 15-second resting period • 3 × 3 -second MVCs, with a 5-second resting period in between • Squat with and without voluntary pre-contraction of the PFMs, with 15-second resting period in between manoeuvres • 1-minute break • 3 × 3 second MVCs, with a 5-second resting period in between • Deadlift with and without voluntary pre-contraction of the PFMs, with 15-second resting period in between manoeuvres Session 2: leg press and curl-up • 5-second resting period • 1 PFM contraction • 15-second resting period • 3 × 3-second MVCs, with 5-second resting period in between • Leg press with and without voluntary pre-contraction of the PFMs, with a 15-second resting period in between manoeuvres • 1-minute break • 3 × 3-second MVCs, with a 5-s resting period in between • Curl-up with and without voluntary pre-contraction of the PFMs, with a 15-s resting period in between manoeuvres	

*PFM* pelvic floor muscle, *MVC* maximal voluntary contractions

## Abbreviations

AI: Anal incontinence
IAP: Intra-abdominal pressure
MVCs: Maximal voluntary contractions
PFD: Pelvic floor disorder
PFM: Pelvic floor muscle
POP: Pelvic organ prolapse
UI: Urinary incontinence
